# Squamous Upper Tract Carcinoma Presenting as a Perinephric Abscess

**DOI:** 10.1155/2013/789097

**Published:** 2013-12-15

**Authors:** M. Lopes, D. Rauber, F. Carvalho, M. Nicodem, J. A. Noronha, G. F. Carvalhal

**Affiliations:** Department of Urology, School of Medicine, Pontifícia Universidade Católica do Rio Grande do Sul, Santo Inácio, 500 apt. 501, 90570-150 Porto Alegre, RS, Brazil

## Abstract

Perinephric abscesses are life-threatening conditions, which are rarely associated with neoplasms of the kidney or upper tract collecting system. We report, to our knowledge, the first case of squamous carcinoma of the upper tract presenting as a perinephric abscess, diagnosed after radical nephrectomy.

## 1. Introduction

Renal and perinephric abscesses are life-threatening conditions, which may occur in different clinical scenarios [1, 2]. Generally, they result from ascending infections complicating an obstructed upper tract. Diabetes mellitus and urolithiasis are important risk factors for these abscesses [3]. Roughly, 30% of all perinephric and renal abscesses are of hematogenic origin, secondary to bacteria located elsewhere in the body (e.g., *Staphylococcus aureus*) [2]. Recently, a new syndrome of renal and perinephric abscesses was associated with infections by methicillin-resistant* Staphylococcus aureus*, in which prompt diagnosis and treatment were key to therapeutic success [4].

Malignant neoplasms are very rarely associated with renal and perirenal abscesses. We report an unusual case of perirenal abscess secondary to a squamous cell carcinoma and discuss the differential diagnosis and therapy.

## 2. Case Presentation

A 82-year-old man presented to the emergency room prostrated, with recent onset of fatigue and malaise. A few days earlier, he started feeling urinary urgency and had started treatment with oral fluoroquinolones for an alleged prostatitis.

Physical examination was unremarkable, there were no abdominal masses, and Giordano's sign was negative. Laboratory revealed mild anemia (Ht: 33%; Hb: 11.3 mg/dL), leukocytosis (29.570 WBCs/mm^3^; 1% myelocytes, 6% band neutrophils), and altered renal function tests (serum creatinine: 2.65 mg/dL; urea: 80 mg/dL). Urinalysis showed leukocyturia (10 leukocytes/field) and hematuria (10 blood cells/field). Urine culture was negative.

A chest X-ray was normal. An abdominal ultrasound showed a 9.6 cm right kidney with poorly defined limits and an internal image suggestive of a choraliform stone. Left kidney was normal, as well as the remainder of the abdominal organs.

The patient was started on IV antibiotics (cefepime) and hydration. After the initial treatment, the patient evolved with worsening of his clinical and laboratory findings. A noncontrasting abdominal and pelvic computerized tomography (CT) revealed a large heterogeneous mass in the right kidney, in contiguity with the lumbar musculature, the liver, and the vena cava, also dislodging the pancreatic head and the duodenum. In the collecting system of the right kidney, a large choraliform stone was found.

**Figure 1:**
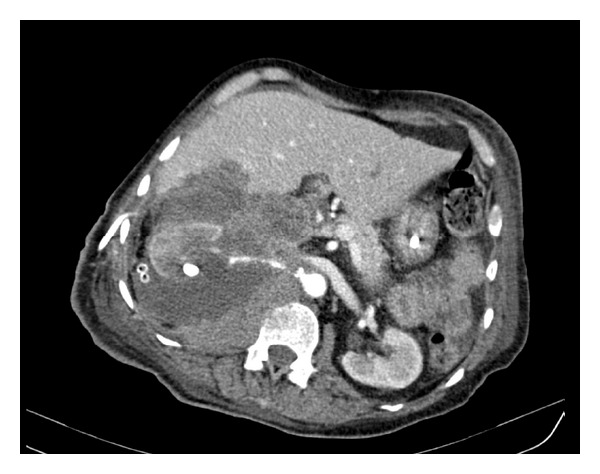
Abdominal computerized tomography with IV contrast, revealing a right kidney with a choraliform stone and a large perirenal abscess.

In spite of the placement of a percutaneous drain in the perirenal space, the patient worsened, with progressive tachycardia, fever, and leukocytosis. Renal function, however, improved (serum Creatinine 1.4 mg/dL). An abdominal CT with intravenous (IV) contrast was then ordered and confirmed a large perirenal abscess (Figure 1), and surgery was indicated. An open radical nephrectomy was performed in the right kidney, which was sent to pathologic examination.

**Figure 2:**
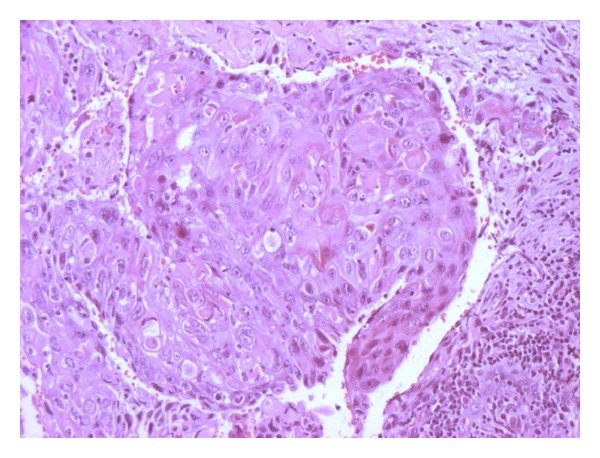
Moderately differentiated (Grade 2) squamous carcinoma of the renal pelvis (H.E., X100).

Pathology revealed an 8.0 cm moderately differentiated (Grade 2) squamous carcinoma of the renal pelvis (Figure 2). Necrosis was present in roughly 30% of the tumor, which extended to the renal parenchyma and the perirenal fat. There was a positive surgical margin in the perihilar region.

The patient survived the perioperative period; however, his clinical condition has not evolved as expected. Roughly one month after the surgical procedure, the patient became septic and died of septic shock of an unknown source. Sepsis was presumed to be urinary, since chest X-rays were normal, and all cultures were negative at the time of death.

## 3. Discussion

Squamous cell cancers are very rare, comprising 0.7% to 7.0% of all urothelial tumors of the upper tract [5, 6]. They generally occur associated with a history of chronic infection or inflammation, which leads to metaplasia of the normal urothelium and to the development of a squamous cell carcinoma [7]. These tumors tend to be moderately to poorly differentiated and are often locally invasive at presentation [6, 8].

The association of renal tumors and perirenal abscesses is very rare. To our knowledge, this is the first published report associating an upper tract squamous cell carcinoma with a perirenal abscess. In our case, the extensive necrosis found in the surgical specimen was probably responsible for the genesis of the perirenal collection, which eventually brought the patient to clinical attention.

The presence of a malignant neoplasm in our case was unsuspected until the time of the radical nephrectomy, and the initial treatment was concerned mostly with the resolution of the perinephric abscess.

We hope that this case report may raise the awareness about the possible association of perirenal abscesses and renal tumors, which may facilitate the diagnosis and therapy of similar occurrences.
